# Inflammation-related parameter serve as prognostic biomarker in esophageal squamous cell carcinoma

**DOI:** 10.3389/fonc.2022.900305

**Published:** 2022-10-21

**Authors:** Xiaoqin Xu, Jiexian Jing

**Affiliations:** Department of Etiology, Shanxi Province Cancer Hospital, Shanxi Hospital Affiliated to Cancer Hospital, Chinese Academy of Medical Sciences, Cancer Hospital Affiliated to Shanxi Medical University, Taiyuan, China

**Keywords:** ESCC, LMR, inflammation, prognosis, marker

## Abstract

**Objective:**

The aim of this study was to explore the predictive role of inflammation-related parameters in prognosis of esophageal squamous cell carcinoma (ESCC).

**Methods:**

A total of 370 ESCC patients subjected to curative surgery were enrolled. All patients had complete medical records and did not receive preoperative adjuvant therapy. Preoperative systemic immune-inflammation index (SII) was calculated as platelet count × neutrophil count/lymphocyte count, prognostic nutrition index (PNI) as albumin concentration (g/L) + 5 × total lymphocyte count (10^9^/L), and systemic inflammation response index (SIRI) as neutrophil count × monocyte count/lymphocyte count. The optimal cut‐off values of preoperative SII, neutrophil‐to‐lymphocyte ratio (NLR), platelet‐to‐lymphocyte ratio (PLR), lymphocyte-to-monocyte ratio (LMR), PNI, and SIRI were determined *via* receiver operating characteristic (ROC) analysis, and their correlations with clinical parameters and survival analyzed.

**Results:**

NLR was associated with gender (*P* = 0.022), and PLR (*P* = 0.037), PNI (*P* = 0.017) was associated with survival status, LMR was related with gender (*P* = 0.034) and survival status (*P* = 0.01), SIRI was correlated with gender (*P* = 0.000), smoking history (*P* = 0.000) and drinking history (*P* = 0.004). Survival analysis indicated that high PLR (*P* = 0.042), low LMR (*P* = 0.001), and low PNI (*P* = 0.007) were predictive of poor prognosis of ESCC. Stratified analysis revealed the prognostic predictor roles of distinct markers in different ESCC subgroups. SII and SIRI were predominantly correlated with the clinical outcome in the lymphatic metastasis subgroup. Further univariate analysis disclosed that T stage, smoking history, lymphatic metastasis, TNM staging, PLR, LMR, and PNI potentially serve as influencing factors(*P* < 0.05). Multivariate analysis identified T stage (*HR* = 1.781, *P* = 0.002), TNM staging (*HR* = 8.617, *P* = 0.001) and LMR (*HR* = 0.504, *P* = 0.001) as independent predictors for outcomes of ESCC.

**Conclusions:**

Low LMR could serve as an independent marker of poor prognosis in patients with ESCC. Inflammation-related markers have distinct predictive roles in ESCC subgroups with different features.

## Introduction

Esophageal squamous cell carcinoma (ESCC) with a malignant clinical course and negative outcome accounts for ~90% of esophageal cancers in China (1–3). While chemotherapy and radiotherapy are partially beneficial for survival outcomes, repeated recurrence and limited therapeutic approaches have led to low 5-year survival rates of ~30% (4). In recent years, emerging immunotherapy has been successfully applied to treat around 30% of solid tumors. However, the effects of immunotherapy in ESCC are yet to be clinically validated. Identification of safe and reliable biomarkers for assessment of the outcomes and effective monitoring of the therapeutic effects is therefore essential to improve survival rates.

Inflammation is known to play a critical role in cancer progression and management. Hanahan et al. identified tumor-promoting inflammation was considered as a cancer hallmark trait (5). Inflammation is an indispensable innate immune response to the disruption of perturbed tissue homeostasis. Chronic inflammatory response predisposes to all stages of tumor development at all stages and may be utilized as a therapeutic tool (6). Accumulating evidence from recent studies indicates that several inflammatory markers have predictive value in the prognosis of cancer. In clinical practice, the systemic inflammatory response is regarded as a low-cost, highly practicable biomarker, commonly evaluated *via* routine blood tests (leukocytes, neutrophils, lymphocytes, monocytes, platelets, et al) and blood biochemical indicators (albumin, C-reactive protein) (7). Inflammation-related parameters, including neutrophil-lymphocyte ratio (NLR), monocyte-lymphocyte ratio (MLR), platelet-lymphocyte ratio (PLR), and C-reactive protein/albumin ratio (CRP/Alb), are associated with tumorigenesis and development and serve as independent prognostic factors for cancer. Compared with the traditional international Federation of Gynecology and Obstetrics (FIGO) staging system, the nomograms integrating systemic inflammation response index (SIRI) can be used to predict the survival of cervical cancer patients more objectively and reliably after radical resection (8). Breast cancer patients with an increased in SIRI > 75% or 25-75% was shown to be associated with poorer overall survival (OS) than that with no SIRI changes (P< 0.001) (9). Preoperative SIRI may be a reliable predictor of OS in postmenopausal patients with operable breast cancer, providing personalized prognostication and assistance in the formulation of clinical treatment strategies (10). Additionally, the systemic immune-inflammation index (SII) is proposed to play a prognostic predictive role in ESCC (11). SIRI has been reported as an independent prognostic index in radically resected ESCC (12). Systemic inflammation score (SIS) is a novel and more promising inflammation-based prognostic tool than the prognostic nutrition index (PNI) in ESCC patients subjected to esophagectomy (13). However, to our best knowledge, available studies based on related inflammatory response indexes in patients with surgically resected ESCC are limited, with controversial results.

In the present study, we further focused on prognostic factors of ESCC and evaluated the prognostic performance of inflammation-related parameters in predicting postoperative survival in our patient cohorts.

## Materials and methods

### Patients

This study was a single-center, retrospective design. In total, 370 ESCC patients were enrolled at Shanxi Province Cancer Hospital between October 2016 and May 2018. None of the subjects had received neoadjuvant chemoradiotherapy or any other cancer treatments before operative treatment. Surgery was performed in all cases and ESCC diagnosis was subsequently confirmed by pathologists. And patients with complete medical information. The following exclusion criteria were applied: (1) patients with other histological types of esophageal carcinoma; (2) patients who had received any EC-related treatment before recruitment; (3) patients whose diagnosis was not been confirmed by pathology; and (4) patients with incomplete medical information. Clinical staging of ESCC was determined based on the 8th edition of the TNM staging system of esophageal cancer by the American Joint Commission on Cancer and the Union for International Cancer Control. Follow-up was performed *via* telephone and outpatient consultation based on National Comprehensive Cancer Network (NCCN) guidelines. OS time was assessed from the date of surgery to the date of the most recent follow-up or death. The follow-up period ended in November 2021.

### Data collection and the definition of inflammation-related parameters

All preoperative blood routine and biochemical results were recorded. Calculations for indicators were performed as follows: NLR = neutrophil count/lymphocyte count, PLR = platelet count/lymphocyte count, LMR = lymphocyte count/monocyte count, SII = platelet count × neutrophil count/lymphocyte count, PNI = albumin concentration (g/L) + 5×total lymphocyte count (10^9^/L), SIRI = neutrophil count × monocyte count/lymphocyte count.

### Statistical analysis

All data were statistically analyzed by employing the SPSS 22.0 software package (IBS SPSS, Armonk, NY, USA). The optimal cut-off values of NLR, PLR, SII, PNI, and SIRI were determined *via* receiver operating characteristic analysis. The association of multiple predictors with clinicopathological features of ESCC was analyzed *via* the chi-square test. Overall survival stratified by multiple inflammatory biomarkers was assessed using the Kaplan–Meier method and log-rank test. Univariate and multivariate analyses were performed based on the Cox proportional hazards regression model. P values less than 0.05 were considered statistically significant.

## Results

### Basic characteristics of patients

The 370 ESCC patients enrolled consisted of 245 males (66.22%) and 125 females (33.78%). The median age was 61 years (40-81 years). In total, 47.84% of ESCC patients had exposure to tobacco consumption, and 29.73% patients harbored an alcohol drinking lifestyle. The primary location was the middle esophagus in 72.43% of cases, the lower segment in 23.51% of cases, and the upper esophagus in only 4.05% cases. Moderately differentiated ESCC accounted for 65.68% of the total cases, followed by poorly differentiated (31.89%) and well-differentiated ESCC (2.43%). Overall, 65.68% of ESCC patients were at the advanced T stage at diagnosis and only a small proportion of patients (32.32%) were diagnosed at the early T stage. In total, 43.78% of ESCC patients harbored advanced TNM stages at the time of diagnosis, and ESCC was accompanied by lymphatic metastasis in 45.41% of cases. The collective clinical, epidemiological, and pathological features are presented in **Table 1**.

**Table 1:** The clinical characteristics of ESCC patients.

Characteristics	n
**Gender**	
Male/Female	245/125
**Age**	
<60years/≥60years	158/212
**Location**	
Upper/Middle/Lower	15/268/87
**Smoking history**	
No/Yes	193/177
**Drinking history**	
No/Yes	260/110
**Grade**	
G1+G2/G3	9/243/118
**TNM staging**	
I/II/III/IV	32/176/135/27
**T stage**	
T1/T2/T3/T4	36/91/241/2
**Lymphaticmetastasis**	
No/Yes	202/168
**Survival status**	
Live/Deceased	191/179

### Associations between NLR, PLR, LMR, PNI, SII, SIRI and relevant clinicopathological characteristics in ESCC patients

We further analyzed the correlations between six inflammation-related parameters and clinicopathological features in ESCC patients. ROC analysis revealed optimum cut-off values of NLR, PLR, LMR, PNI, SII, SIRI of 1.304, 98.7144, 3.1049, 50.6, 313.6978, and 0.4854, respectively. Accordingly, patients were divided into two groups with low or high levels of each parameter based on the optimal cutoff value for further correlation analysis. As shown in **Table 2**, NLR was associated with gender (*χ2* = 5.255, *P* = 0.022), PLR (*χ2* = 4.369, *P* = 0.037) and PNI (*χ2* = 5.717, *P* = 0.017) with survival status, LMR with gender (*χ2* = 4.503, *P* = 0.034) and survival status (*χ2* = 6.686, *P* = 0.01), and SIRI with gender (*χ2* = 14.316, *P* = 0.000), smoking history (*χ2* = 12.709, *P* = 0.000) and drinking history (*χ2* = 8.078, *P* = 0.004). SII was not associated with all clinical and pathological parameters. The collective results support an association of the inflammation-related index with clinical features of ESCC.

**Table 2:** The relationship between inflammatory parameter and clinical pathological features.

** **	NLR		** **		PLR				SII		
	Low	High	*χ^2^*	*P*	Low	High	*χ^2^*	*P*	Low	High	*χ^2^*	*P*
	(n = 55)	(n = 315)			(n = 99)	(n = 271)			(n = 94)	(n = 276)		
**Gender**												
Male	29 (11.8)	216 (88.2)	5.255	0.022*	71 (29.0)	174 (71.0)	1.828	0.176	62 (25.3)	183 (74.7)	0.004	0.951
Female	26 (20.8)	99 (79.2)			28 (22.4)	97 (77.6)			32 (25.6)	93 (64.4)		
**Age**												
<60years	21 (13.3)	137 (86.7)	0.540	0.463	39 (24.7)	119 (75.3)	0.605	0.437	43 (27.2)	115 (72.8)	0.477	0.490
≥60years	34 (16.0)	178 (84.0)			60 (28.3)	152 (71.7)			51 (24.1)	161 (75.9)		
**Location**												
Upper	2 (13.3)	13 (86.7)	1.104	0.576	2 (13.3)	13 (86.7)	1.484	0.476	3 (20.0)	12 (80.0)	1.728	0.422
Middle	43 (16.0)	225 (84.0)			74 (27.6)	194 (72.4)			73 (27.2)	195 (72.8)		
Lower	10 (11.5)	77 (88.5)			23 (26.4)	64 (73.6)			18 (20.7)	69 (79.3)		
**Smoking history**												
No	35 (18.1)	158 (81.9)	3.409	0.065	45 (23.3)	148 (76.7)	2.437	0.118	46 (23.8)	147 (76.2)	0.526	0.468
Yes	20 (11.3)	157 (88.7)			54 (30.5)	123 (69.5)			48 (27.1)	129 (72.9)		
**Drinking history**												
No	44 (16.9)	216 (83.1)	2.927	0.087	70 (26.9)	190 (73.1)	0.012	0.912	70 (26.9)	190 (73.1)	1.063	0.303
Yes	11 (10.0)	99 (90.0)			29 (26.4)	81 (73.6)			24 (21.8)	86 (78.2)		
**Grade**												
G1+G2	40 (15.9)	212 (84.1)	0.635	0.426	72 (28.6)	180 (71.4)	1.328	0.249	66 (26.2)	186 (73.8)	0.257	0.612
G3	15 (12.7)	103 (87.3)			27 (22.9)	91 (77.1)			28 (23.7)	90 (76.3)		
**TNM staging**												
I+II	35 (16.8)	173 (83.2)	1.445	0.229	62 (29.8)	146 (70.2)	2.256	0.133	53 (25.5)	155 (74.5)	0.001	0.970
III+IV	20 (12.3)	142 (87.7)			37 (22.8)	125 (77.2)			41 (25.3)	121 (74.7)		
**T stage**												
T1+T2	23 (18.3)	104 (81.7)	1.609	0.205	38 (29.9)	89 (70.1)	0.988	0.320	36 (28.3)	91 (71.7)	0.883	0.347
T3+T4	32 (13.2)	211 (86.8)			61 (25.1)	182 (74.9)			58 (23.9)	185 (76.1)		
**Lymphatic metastasis**												
No	32 (15.8)	170 (84.2)	0.335	0.563	58 (28.7)	144 (71.3)	0.869	0.351	49 (24.3)	153 (85.7)	0.309	0.578
Yes	23 (13.7)	145 (86.3)			41 (24.4)	127 (75.6)			45 (26.8)	123 (83.2)		
**Survival status**												
Live	33 (17.3)	158 (82.7)	1.816	0.178	60 (31.4)	131 (68.6)	4.369	0.037*	54 (28.3)	137 (71.7)	1.712	0.191
Deceased	22 (12.3)	157 (87.7)			39 (21.8)	140 (78.2)			40 (22.3)	139 (77.7)		
	**PNI**				**LMR**				**SIRI**			
	**Low**	**High**			**Low**	**High**			**Low**	**High**		
	**(n = 95)**	**(n = 275)**	**χ*2***	***P***	**(n = 56)**	**(n = 314)**	**χ*2***	***P***	**(n = 82)**	**(n = 288)**	**χ*2***	***P***
**Gender**												
Male	63 (25.7)	182 (74.3)	0.001	0.981	44 (18.0)	201 (82.0)	4.503	0.034*	40 (16.3)	205 (83.7)	14.316	0.000***
Female	32 (25.6)	93 (74.4)			12 (9.6)	113 (90.4)			42 (33.6)	83 (66.4)		
**Age**												
<60years	37 (23.4)	121 (76.6)	0.737	0.791	29 (12.7)	98 (87.3)	1.317	0.251	33 (20.9)	125 (79.1)	0.260	0.610
≥60years	58 (27.4)	154 (72.6)			36 (17.0)	135 (83.0)			49 (23.1)	163 (76.9)		
**Location**												
Upper	5 (33.3)	10 (66.7)	1.226	0.542	1 (6.7)	14 (93.3)	2.343	0.310	3 (20.0)	12 (80.0)	3.613	0.164
Middle	71 (26.5)	197 (73.5)			38 (14.2)	230 (85.8)			66 (24.6)	202 (75.4)		
Lower	19 (21.8)	68 (78.2)			17 (19.5)	70 (80.5)			13 (14.9)	74 (85.1)		
**Smoking history**												
No	54 (28.0)	139 (72.0)	1.122	0.290	31 (16.1)	162 (83.9)	0.270	0.603	57 (29.5)	136 (70.5)	12.709	0.000***
Yes	41 (23.2)	136 (76.8)			25 (14.1)	152 (85.9)			25 (14.1)	152 (85.9)		
**Drinking history**												
No	67 (25.8)	193 (74.2)	0.004	0.950	39 (15.0)	221 (85.0)	0.012	0.911	68 (26.2)	192 (73.8)	8.078	0.004**
Yes	28 (25.5)	82 (74.5)			17 (15.5)	93 (84.5)			14 (12.7)	96 (87.3)		
**Grade**												
G1+G2	64 (25.4)	188 (74.6)	0.032	0.858	34 (13.5)	218 (86.5)	1.661	0.197	58 (23.0)	194 (77.0)	0.334	0.563
G3	31 (26.3)	87 (73.7)			22 (18.6)	96 (81.4)			24 (20.3)	94 (79.7)		
**TNM staging**												
I+II	48 (23.1)	160 (76.9)	1.681	0.195	29 (13.9)	179 (86.1)	0.526	0.468	49 (23.6)	159 (75.2)	0.536	0.464
III+IV	47 (29.0)	115 (71.0)			27 (16.7)	135 (83.3)			33 (20.4)	129 (79.6)		
**T stage**												
T1+T2	28 (22.0)	99 (78.0)	1.334	0.248	20 (15.7)	68 (84.3)	0.057	0.812	29 (22.8)	98 (77.2)	0.051	0.822
T3+T4	67 (27.6)	176 (62.4)			36 (14.8)	152 (85.2)			53 (21.8)	190 (78.2)		
**Lymphatic metastasis**												
No	47 (23.3)	155 (76.7)	1.352	0.245	28 (13.9)	174 (86.1)	0.562	0.453	45 (22.3)	157 (77.7)	0.003	0.953
Yes	48 (28.6)	120 (71.4)			28 (16.7)	140 (83.3)			37 (22.0)	131 (78.0)		
**Survival status**												
Live	39 (20.4)	152 (79.6)	5.717	0.017*	20 (10.5)	171 (89.5)	6.686	0.010*	47 (24.6)	144 (75.4)	1.368	0.242
Deceased	56 (31.3)	123 (68.7)			36 (20.1)	143 (79.9)			35 (19.6)	144 (80.4)		

*: *P* < 0.05, **: *P* < 0.01, ***: *P* < 0.001.

### Higher PLR, lower LMR, and lower PNI are predictive factors in the survival of patients with ESCC

**Figure 1:**
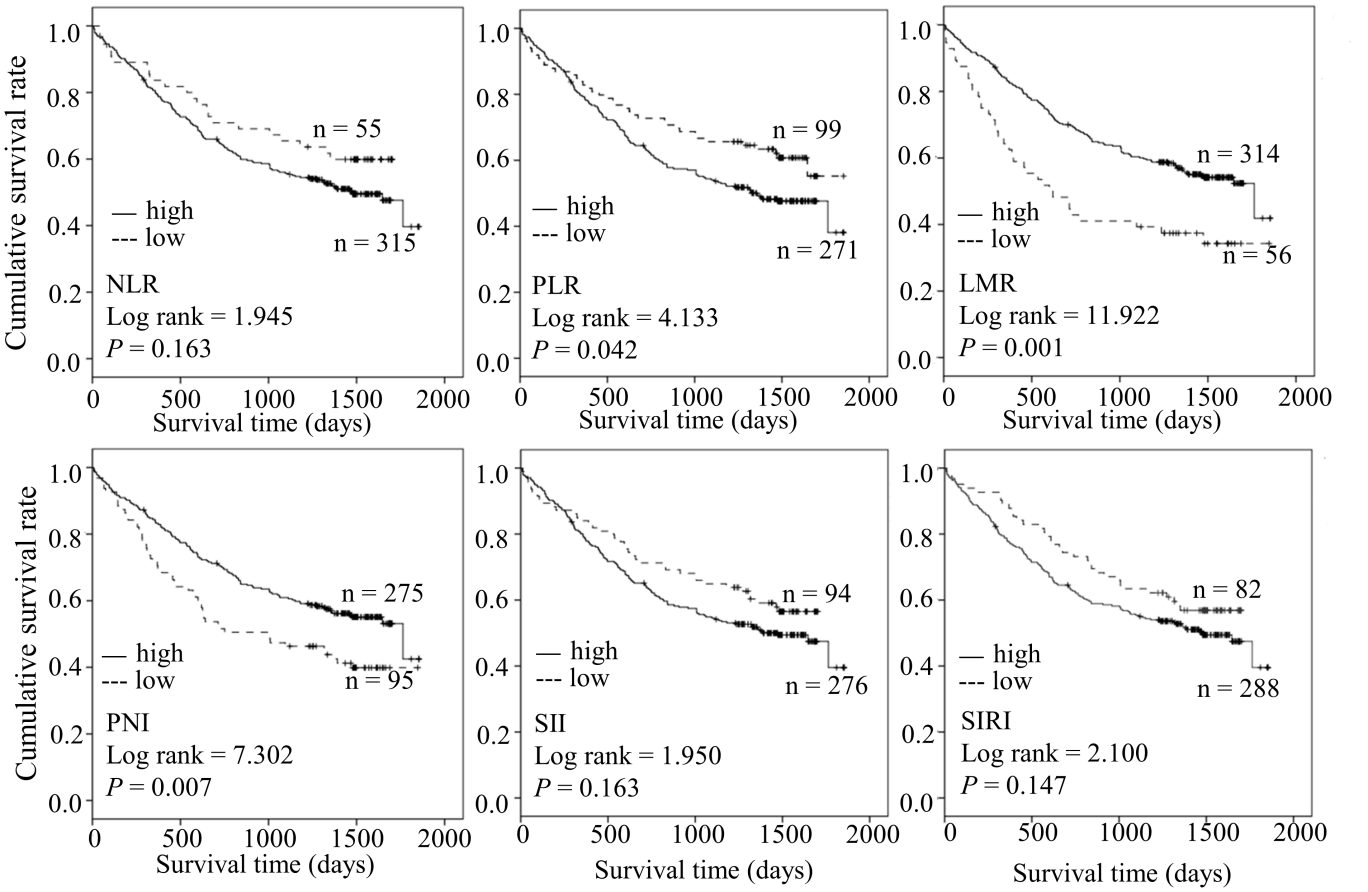
The prognosis predicting role of preoperative NLR, PLR, LMR, SII, SIRI, PNI.

We further compared the difference in OS between the two groups. Kaplan-Meier analysis revealed shorter OS in the high PLR group relative to in the low PLR group (log-rank = 4.133, *P* = 0.042). Moreover, ESCC cases with low LMR(log-rank = 11.922, *P* = 0.001) and low PNI (log-rank = 7.302, *P* = 0.007) had poorer prognosis. Our results suggest that PLR, LMR, and PNI collectively serve as predictive factors of survival in ESCC (**Figure 1**).

### Preoperative inflammatory markers are potentially associated with adverse clinical outcomes in combination with diverse features of ESCC

**Figure 2:**
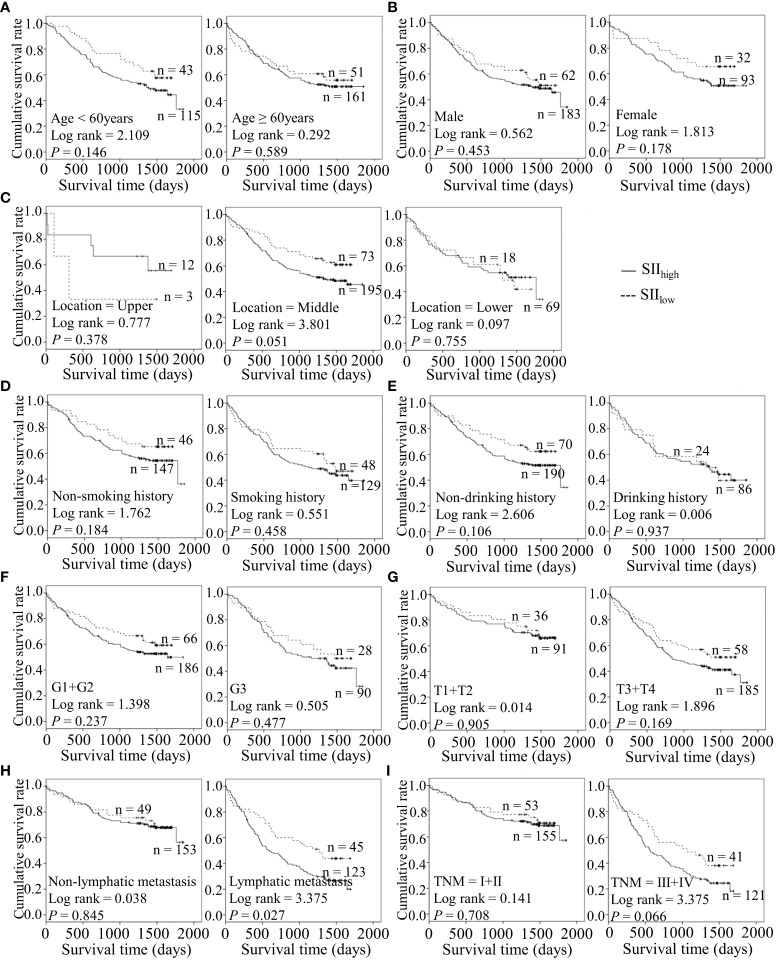
Predicating role of preoperative SII level for the OS of ESCC patients. **(A–I)** Kaplan-Meier survival curves of ESCC patients with different SII levels combined with diverse features like age **(A)**, gender **(B)**, tumor location **(C)**, smoking history **(D)**, drinking history **(E)**, tumor grade **(F)**, T stage **(G)**, lymph node metastasis **(H)** and TNM staging **(I)**.

**Figure 3:**
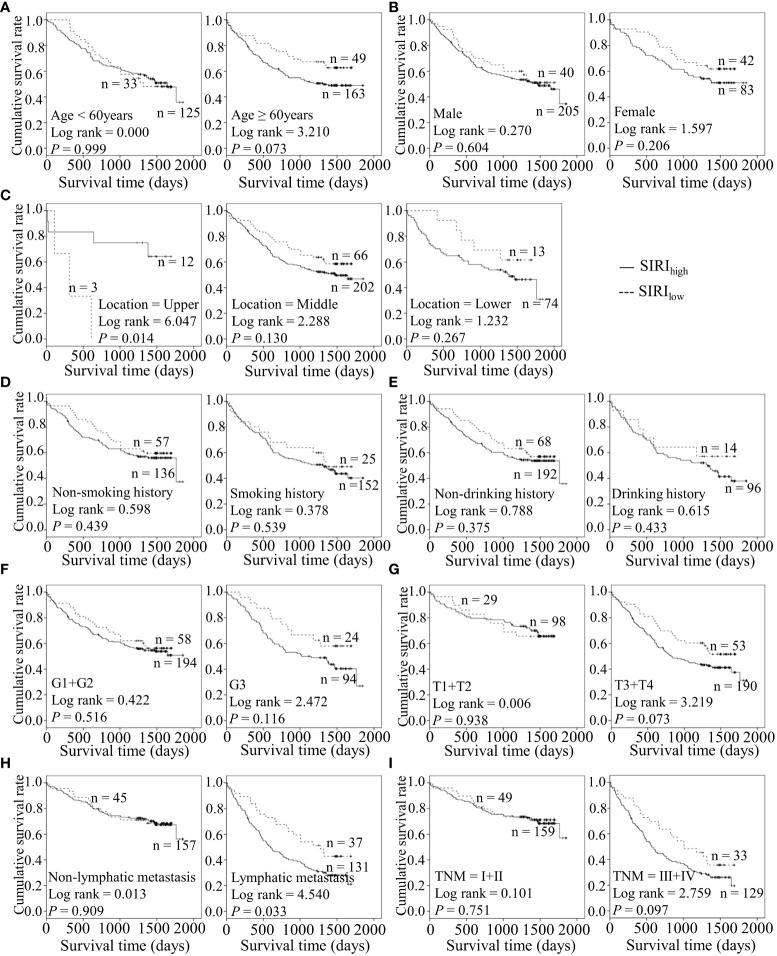
Predicating role of preoperative SIRI level for the OS of ESCC patients. **(A–I)** Kaplan-Meier survival curves of ESCC patients with different SII levels combined with diverse features like age **(A)**, gender **(B)**, tumor location **(C)**, smoking history **(D)**, drinking history **(E)**, tumor grade **(F)**, T stage **(G)**, lymph node metastasis **(H)** and TNM staging **(I)**.

**Figure 4:**
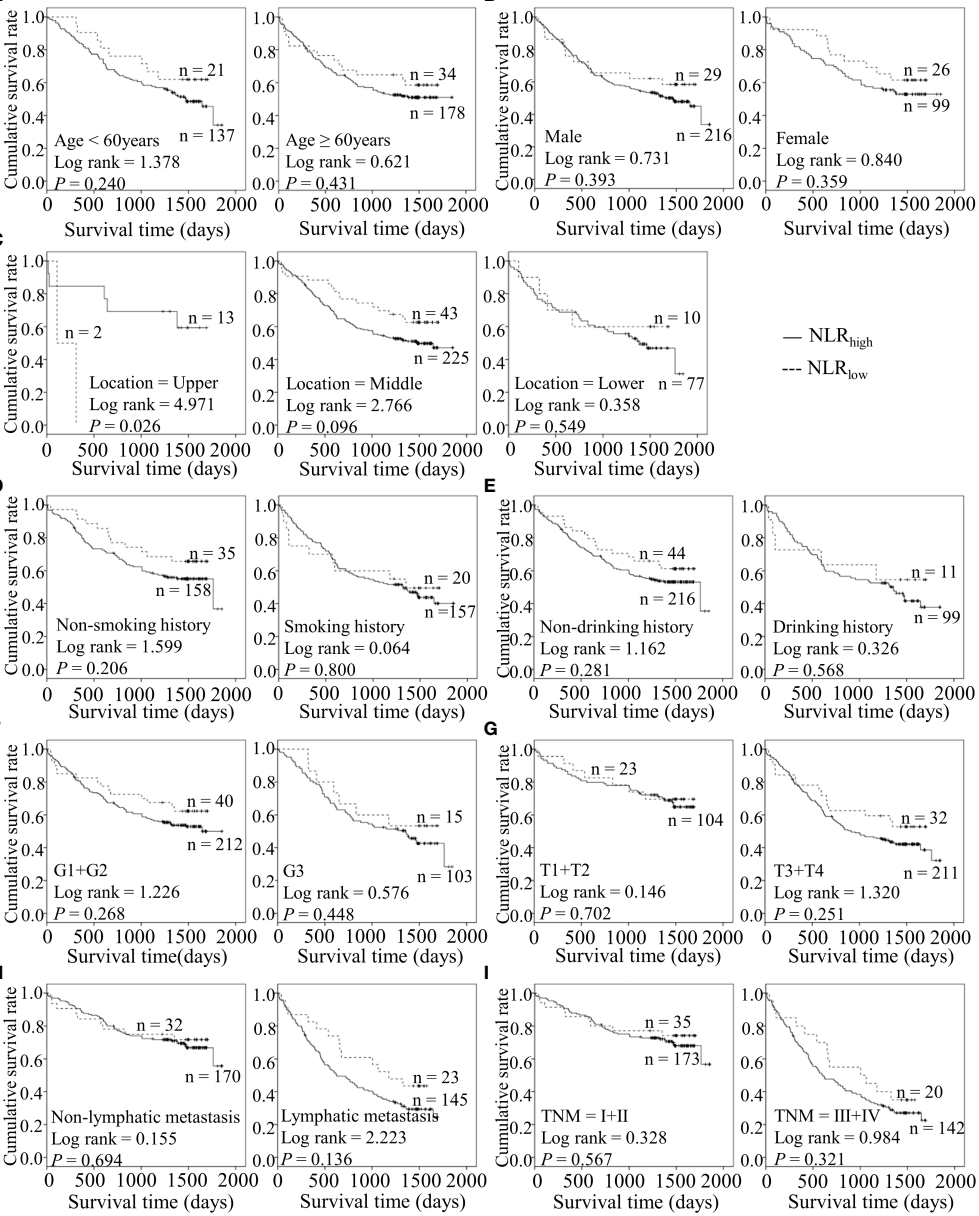
Predicating role of preoperative NLR level for the OS of ESCC patients. **(A–I)** Kaplan-Meier survival curves of ESCC patients with different NLR levels combined with diverse features like age **(A)**, gender **(B)**, tumor location **(C)**, smoking history **(D)**, drinking history **(E)**, tumor grade **(F)**, T stage **(G)**, lymph node metastasis **(H)** and TNM staging **(I)**.

**Figure 5:**
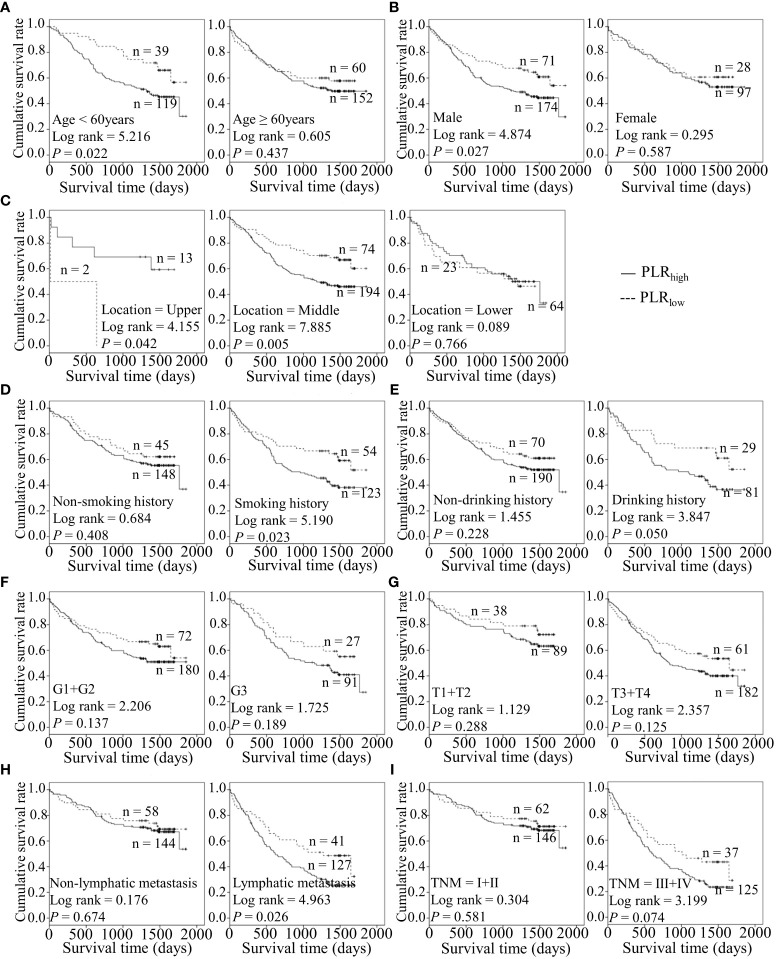
Predicating role of preoperative PLR level for the OS of ESCC patients. **(A–I)** Kaplan-Meier survival curves of ESCC patients with different PLR levels combined with diverse features like age **(A)**, gender **(B)**, tumor location **(C)**, smoking history **(D)**, drinking history **(E)**, tumor grade **(F)**, T stage **(G)**, lymph node metastasis **(H)** and TNM staging **(I)**.

**Figure 6:**
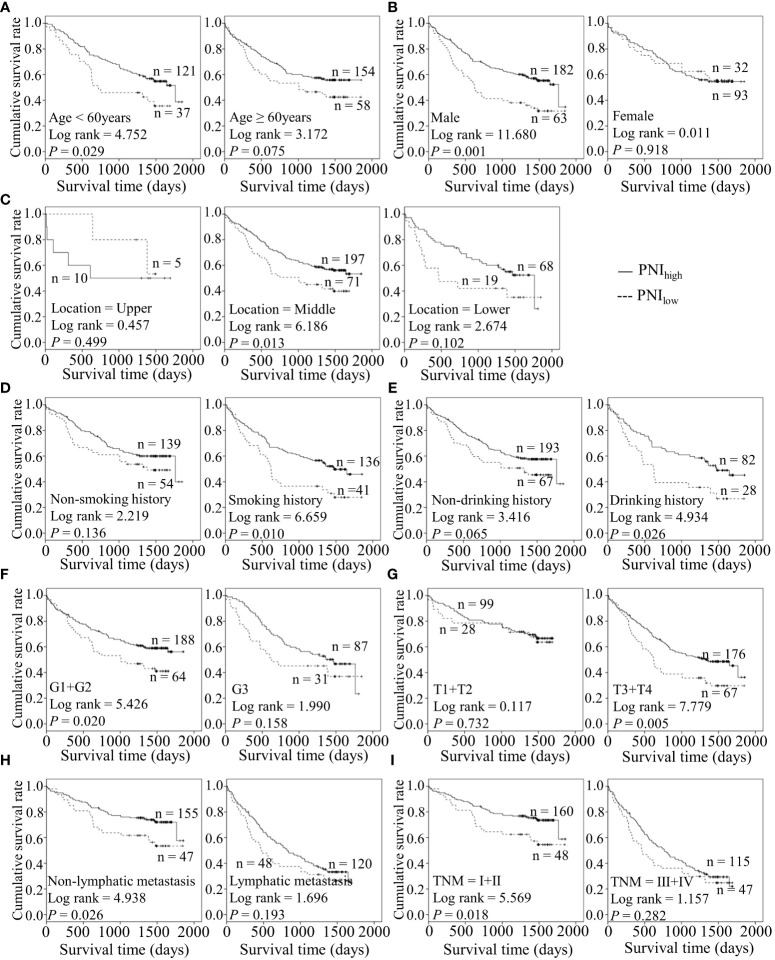
Predicating role of preoperative PNI level for the OS of ESCC patients. **(A–I)** Kaplan-Meier survival curves of ESCC patients with different PNI levels combined with diverse features like age **(A)**, gender **(B)**, tumor location **(C)**, smoking history **(D)**, drinking history **(E)**, tumor grade **(F)**, T stage **(G)**, lymph node metastasis **(H)** and TNM staging **(I)**.

**Figure 7:**
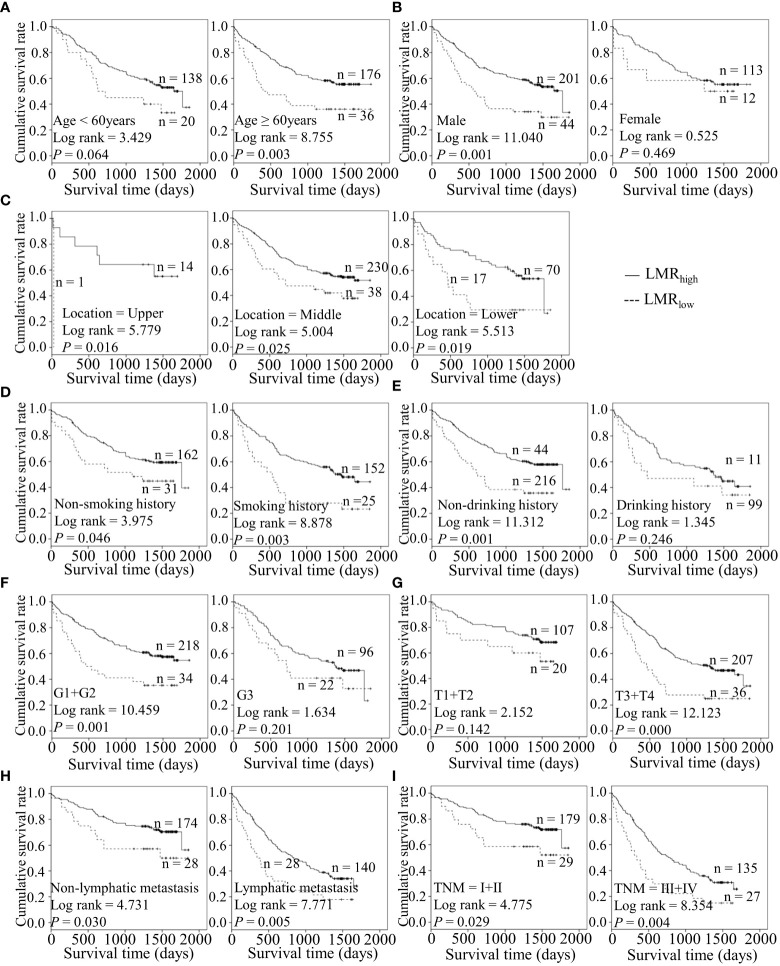
Predicating role of preoperative LMR level for the OS of ESCC patients. **(A–I)** Kaplan-Meier survival curves of ESCC patients with different LMR levels combined with diverse features like age **(A)**, gender **(B)**, tumor location **(C)**, smoking history **(D)**, drinking history **(E)**, tumor grade **(F)**, T stage **(G)**, lymph node metastasis **(H)** and TNM staging **(I)**.

Considering the predictive prognostic role of inflammatory parameters, a stratified analysis was conducted to further determine the high-risk patient group. In terms of SII index, higher SII was associated with poorer prognosis than the control group when stratified by level of lymphatic metastasis (**Figure 2**). Similarly, among ESCC patients with lymphatic metastasis, shorter OS was observed for the SIRI_low_ group than the SIRI_low_ group (**Figure 3**). However, the SIRI_low_ group had shorter OS than the SIRI_high_ group among patients with upper ESCC. NLR was specifically associated with OS in the upper ESCC subgroup (**Figure 4**). These findings support the utility of higher SII and SIRI as prognosis predictors in ESCC cases with lymphatic metastasis. Moreover, higher PLR was associated with poorer clinical outcomes for the following groups of ESCC patients: age < 60 years, male, smoker, and lymphatic metastasis (**Figure 5**). Interestingly, the poor prognosis was associated with upper ESCC harboring lower PLR while opposite results were obtained for the middle ESCC group. PNI showed superior predictive ability at all group levels after stratification, especially in the following groups of patients: age < 60 years, male, middle location, smokers, drinkers, G1+G2, advanced T staging, non-lymphatic metastasis, and early TNM stage. Our data indicate that preoperative PNI is a useful predictor of OS in ESCC patients (**Figure 6**). As shown in **Figure 7**, lower LMR may have prognostic predictive value in specific groups of patients (aged ≥ 60 years, male, non-drinkers, G1+G2, and advanced T staging). Overall, the predictive capability of the six parameters was associated with different features of ESCC which should facilitate the identification of high-risk populations for clinical management.

### LMR serves as an independent predictor for the prognosis of ESCC

To further validate the predictive role of inflammation-related parameters in the survival status of ESCC patients, univariate and multivariate Cox proportional hazard regression analyses were performed. Univariate analysis revealed that T stage (*HR* = 2.098, *95% CI* = 1.483–2.969, *P* = 0.000), smoking history (*HR* = 1.382, *95% CI* = 1.03–1.855, *P* = 0.031), lymphatic metastasis (*HR* = 3.708, *95% CI* = 2.259–4.193, *P* = 0.000), TNM staging (*HR* = 3.530, *95% CI* = 2.586–4.818, *P* = 0.000), and PLR (*HR* = 1.442, *95% CI* = 1.011–2.058, *P* = 0.043) may enhance survival risk of ESCC while LMR (*HR* = 0.53, *95% C*I = 0.368–0.765, *P* = 0.001) and PNI (*HR* = 0.649, *95% CI* = 0.473–0.890, *P* = 0.007) were identified as protective factors (**Table 3**). In multivariate analysis, T stage (*HR* = 1.781, *95%CI* = 1.245–2.548, *P* = 0.002), TNM staging (*HR* = 8.617, *95% CI* = 2.43–30.551, *P* = 0.001) and LMR (*HR* = 0.504, *95% CI* = 0.331–0.768, *P* = 0.001) were identified as an independent predictors of adverse events in ESCC (**Table 3**). Importantly, T stage and TNM staging appear to be risk factors and preoperative LMR plays a protective role in survival outcomes of ESCC patients subjected to surgery. Our findings support the utility of LMR as a superior predictor of ESCC survival outcomes.

**Table 3:** The univariate and multivariate analysis.

	Univariate			Multivariate		
	*HR*	*P*	*95%CI*	*HR*	*P*	*95%CI*
Age	1.040	0.795	0.774-1.397	1.078	0.641	0.787-1.475
Gender	0.831	0.250	0.605-1.139	1.096	0.676	0.712-1.688
Location						
Upper		0.896			0.267	
Middle	0.962	0.920	0.449-2.059	0.616	0.231	0.278-1.361
Lower	1.044	0.917	0.470-2.318	0.516	0.122	0.224-1.192
Grade	1.236	0.173	0.911-1.677	1.116	0.494	0.815-1.529
T stage	2.098	0.000***	1.483-2.969	1.781	0.002**	1.245-2.548
Smoking history	1.382	0.031*	1.030-1.855	1.446	0.116	0.913-2.288
Drinking history	1.291	0.106	0.947-1.759	1.037	0.855	0.704-1.528
Lymph node metastasis	3.078	0.000***	2.259-4.193	0.390	0.142	0.111-1.371
TNM staging	3.716	0.000***	2.726-5.064	8.617	0.001**	2.430-30.551
NLR	1.372	0.165	0.878-2.145	0.845	0.559	0.480-1.488
PLR	1.442	0.043*	1.011-2.058	1.050	0.828	0.678-1.626
SII	1.284	0.164	0.903-1.826	1.103	0.700	0.670-1.817
LMR	0.530	0.001**	0.368-0.765	0.504	0.001**	0.331-0.768
PNI	0.649	0.007**	0.473-0.890	0.779	0.164	0.547-1.108
SIRI	1.313	0.149	0.907-1.901	1.180	0.472	0.751-1.856

*: *P* < 0.05, **: *P* < 0.01, ***: *P* < 0.001.

## Discussion

In the present study, we examined the predictive value of preoperative inflammation-related parameters and evaluated their clinical significance in ESCC. A number of index-based routine blood parameters including NLR, PLR, LMR, SII, SIRI, and PNI, were correlated with specific malignant characteristics of ESCC.

NLR was significantly associated with gender (88.2% male ESCC patients), consistent with the finding of Gao Y et al. (11). In terms of the epidemiology of ESCC, male patients accounted for 70% of esophageal cancer cases, with 2- to 3-fold differences in incidence and mortality rates between the genders (14). Similarly, LMR and SIRI were correlated with gender, in keeping with the other available studies (12, 15), suggesting that these indicators are associated with an elevated risk of ESCC. Our analysis additionally revealed correlations of SIRI with smoking and drinking history. Epidemiologically, tobacco smoking and alcohol consumption have been identified as critical risk factors for ESCC in economically developed countries (16), exerting variable carcinogenic effects based on the degree of consumption. Unexpectedly, SII was not associated with all clinical and pathological features of 370 ESCC patients in this study, possibly due to the limited sample size and heterogeneity. We observed that lower LMR and PNI, and higher PLR were associated with increased deaths. LMR and PNI appeared negatively correlated while PLR was positively correlated with survival status. Further, survival analysis revealed shorter OS of high PLR, low LMR, and low PNI groups, suggesting that the three biomarkers are associated with unfavorable clinical outcomes, in keeping with earlier literature (15, 17, 18). However, the complex and dynamic mechanisms by which these indicators promote carcinogenesis are yet to be established.

The tumor environment is the dominant setting that fosters tumorigenesis and development (19). In addition to immune cells and molecular, infiltrating inflammatory cells are clearly involved in tumor behavior. Notably, systemic or local inflammation can facilitate tumor formation and progression in an inflammatory cell-dependent manner. Thus, neutrophils, platelets, lymphocytes, and monocytes derived from peripheral blood provide a causal link between inflammation and cancer. Considerable clinical evidence has highlighted the contribution of these cells to tumor progression and their predicting prognostic value in various cancer types (20). Since the late 1980s, a decisive role of neutrophils in primary tumor growth through entry into the tumor microenvironment and interactions with cancer cells has been reported (21). Mechanistically, recruited neutrophils express inducible nitric oxide synthase(iNOS) and arginase 1 (ARG1) to inhibit activation and anti-tumor effects of CD8 ^+^ T cells (12, 21–23), produce reactive oxygen species (ROS) or reactive nitrogen species (RNS) to facilitate the transformation of epithelial to cancer cells, and induce BV8 and vascular endothelial growth factor (VEGF), leading to remodeling of extracellular matrix (ECM) and mediation of angiogenesis (22, 23). Additionally, neutrophils play an essential role in expediting metastasis by releasing neutrophil extracellular traps (NET). Generally, circulating lymphocytes modulate tumor growth and improve the survival rates of cancer patients through the production of cytokines (IFN-γ and TNF-α), and thus reduced quantity or exhaustion of lymphocytes impairs immune surveillance and defense in cancer (24, 25). Recent findings indicate that tumor-associated macrophages (TAM) generated from monocytes affect tumorigenesis. Increased infiltration of TAM is associated with the survival status of various cancers. Hence, the number and percentage of monocytes could substitute for TAMs to mirror the tumor burden. Notably, LMR may reflect the dynamic balance between the pro-tumor reaction and anti-tumor immune response and therefore be significantly associated with adverse clinical prognosis. Additionally, platelets are considered active players in stimulating tumorigenesis and hematogenous metastatic dissemination by recruiting myeloid cells, secreting platelet-secreted growth factors, and specific chemokines (26, 27). Thrombocytosis in cancer patients is associated with adverse patient survival. Our results confirmed an association of high PLR with a poorer prognosis of cancer. Given their rich microparticle and exosome contents, platelets are well-positioned to coordinate the interplay between tumors and hosts.

We further conducted stratified analysis for identifying the high-risk populations for clinical management. Previous studies suggested that lymph node metastasis can commonly occur in ESCC and is predictive of a poorer prognosis (28, 29). Both SII and SIRI showed utility in prognostic prediction in ESCC patients with lymphatic metastasis. These two factors may therefore serve as valuable lymphatic metastasis-related prognosis markers for ESCC. Nevertheless, NLR was less powerful for all subgroups except upper ESCC, suggesting a correlation with tumor location. Primary locations of esophageal tumors are reported to be correlated with distant metastasis sites (30). For example, patients with upper esophageal cancer are prone to lung metastasis while lower ESCC is more commonly associated with liver invasion. Given the limited number of cases of upper ESCC, further studies on location-related ESCC are essential. Additionally, we examined the specific roles of PLR, LMR, and PNI in the prognosis of different ESCC subgroups. All three markers showed significant association with the male gender. Lower LMR was correlated with shorter OS in older ESCC patients, while higher PLR and lower PNI were predictive of poor prognosis in young patients, supporting their use as age-related prognostic markers. Higher PLR was more relevant to deceased status for upper and the middle subgroups, and lower PNI correlated with a tendency of shorter OS for the middle ESCC subgroup. These associations with location-related metastasis patterns require further exploration.

Tobacco smoking and alcohol are lifestyle risk factors for ESCC (16). Recent research suggests that these lifestyle habits can accelerate the progressive age-related expansion of clones and remodel the esophageal epithelium driven by mutation (31). Higher PLR was predictive of poorer outcomes in ESCC with positive smoking status and lower PNI played a predictive role in cases with exposure to tobacco and alcohol in our experiments. Conversely, lower LMR was correlated with poor prognosis for patients with no drinking history. For well and moderately differentiated and advanced T staging groups, lower LMR, and PNI had better predictive values. Importantly, lower PNI was related to survival in the ESCC subgroup at the early TNM stage and in the absence of lymphatic metastasis. The data suggest that pre-surgical PNI has good performance in assessing the prognosis of the early diagnosis subgroup of ESCC patients. Therefore, these different markers appear to exert variable effects in different ESCC subgroups.

Finally, we screened the independent risk factors were screened *via* Cox regression analysis. Univariate analysis revealed that T stage, smoking history, lymphatic metastasis, TNM staging, and PLR are related to increased risk of ESCC while LMR and PNI serve as protective factors. Furthermore, multivariate analysis showed a 1.717-fold, 5.65-fold, and 0.511-fold risk of adverse events in relation to advanced T stage, TNM staging, and high level of LMR of ESCC. Based on the data, we propose that advanced T stage and TNM staging serve as risk factors and preoperative high LMR had a protective effect on survival outcomes of ESCC following surgery. The collective results indicate that tumor-infiltrating lymphocytes and monocytes play active roles in cancer progression. In our study, we can’t find the superiority of SII and SIRI for OS was not observed, which could be potentially attributed to the limited cohort size and insufficient follow-up period.

Our study has a number of limitations that should be acknowledged. Firstly, this was a single-center retrospective design. Studies on larger sample sizes and avoidance of selection bias *via* multi-center investigations are therefore necessary. Secondly, longer follow-up is essential for the accurate assessment of clinical applications when grouped by different post-surgical therapy. A comprehensive evaluation of the mechanisms underlying the interplay between both these cell types and cancer should be further conducted to facilitate the development of promising treatments targeting cancer-associated inflammation.

## Conclusion

Pretreatment peripheral parameters such as high PLR, low LMR, and low PNI are significantly correlated with adverse events in ESCC. SII and SIRI showed superiority in the prediction of outcomes, especially for the lymphatic metastasis subgroup. Furthermore, LMR may serve as an independent prognostic predictor of long-term survival for patients with ESCC. Given the simplicity, reproducibility, and low cost of analysis of inflammation-related parameters, immune cells present promising indicators of survival in the clinical management of ESCC.

## Data availability statement

The raw data supporting the conclusions of this article will be made available by the authors, without undue reservation.

## Ethics statement

The studies involving human participants were reviewed and approved by Medical Ethics Committee of Shanxi Province Cancer Hospital. The ethics committee waived the requirement of written informed consent for participation.

## Author contributions

XX performed the analysis and wrote the manuscript. JJ supervised the data analysis and revised the manuscript. All authors contributed to the article and approved the submitted version.

## Conflict of interest

The authors declare that the research was conducted in the absence of any commercial or financial relationships that could be construed as a potential conflict of interest.

## Publisher’s note

All claims expressed in this article are solely those of the authors and do not necessarily represent those of their affiliated organizations, or those of the publisher, the editors and the reviewers. Any product that may be evaluated in this article, or claim that may be made by its manufacturer, is not guaranteed or endorsed by the publisher.
